# Protease Activated Receptor-2 Contributes to Heart Failure

**DOI:** 10.1371/journal.pone.0081733

**Published:** 2013-11-27

**Authors:** Silvio Antoniak, Erica M. Sparkenbaugh, Michael Tencati, Mauricio Rojas, Nigel Mackman, Rafal Pawlinski

**Affiliations:** 1 UNC McAllister Heart Institute, Department of Medicine, University of North Carolina at Chapel Hill, Chapel Hill, North Carolina, United States of America; 2 Department of Microbiology, University of Illinois at Urbana-Champaign, Urbana-Champaign, Illinois, United States of America; University of Western Ontario, Canada

## Abstract

Heart failure is a major clinical problem worldwide. Previous studies have demonstrated an important role for G protein-coupled receptors, including protease-activated receptors (PARs), in the pathology of heart hypertrophy and failure. Activation of PAR-2 on cardiomyocytes has been shown to induce hypertrophic growth in vitro. PAR-2 also contributes to myocardial infarction and heart remodeling after ischemia/reperfusion injury. In this study, we found that PAR-2 induced hypertrophic growth of cultured rat neonatal cardiomyocytes in a MEK1/2 and p38 dependent manner. In addition, PAR-2 activation on mouse cardiomyocytes increased expression of the pro-fibrotic chemokine MCP-1. Furthermore, cardiomyocyte-specific overexpression of PAR-2 in mice induced heart hypertrophy, cardiac fibrosis, inflammation and heart failure. Finally, in a mouse model of myocardial infarction induced by permanent ligation of the left anterior descending coronary artery, PAR-2 deficiency attenuated heart remodeling and improved heart function independently of its contribution to the size of the initial infarct. Taken together, our data indicate that PAR-2 signaling contributes to the pathogenesis of hypertrophy and heart failure.

## Introduction

Heart failure (HF) is defined as the failure of the heart to provide the metabolic needs of tissues [1]. It is a major clinical problem of the Western world [2]. In the United States alone, HF results in more than 500,000 deaths per year [2]. HF reflects the end point of both acute and chronic insults, including coronary artery disease, myocardial infarction, hypertension, valve abnormalities and inherited mutations in sarcomere and cytoskeletal proteins [3]–[5].

The major process that contributes to HF is pathologic remodeling of the heart caused by cardiomyocyte hypertrophy, proliferation of cardiac fibroblasts and cardiac inflammation [3], [5]. Cardiomyocytes are generally thought not to proliferate after birth, but can increase in size via hypertrophic growth [4]. Further, cardiac fibroblasts proliferate and synthesize extracellular matrix that contributes to cardiac fibrosis [3]. Depending on the heart disease etiology, different forms of fibrosis can be observed, including perivascular and interstitial fibrosis, as well as deposition of collagen-rich scar tissue at sites of myocardial infarction [3]. Aside from collagen deposition, dysregulated extracellular matrix turnover, orchestrated by the matrix metalloproteinase (MMP)/tissue inhibitor of MMPs (TIMP) system, mediates cardiac fibrosis leading to HF [6]. Cardiac fibrosis and HF severity was further connected to increased levels of inflammatory cytokines and chemokines, such as interleukin (IL)-6 and monocyte chemotactic protein-1 (MCP-1, CCL2) [7]–[10].

Protease activated receptors (PARs) are a family of seven transmembrane domain G protein–coupled receptors activated by proteolytic cleavage [11]. After their activation, a new amino terminus peptide is exposed that functions as a tethered ligand [11]. The PAR family consists of four members: PAR-1, PAR-2, PAR-3, and PAR-4. The coagulation protease thrombin is the main physiological activator of PAR-1, PAR-3 and PAR-4 [11]. PAR-2 is activated by various proteases, including trypsin, mast cells tryptase, and the coagulation proteases FVIIa and FXa [11]–[13]. PARs can be also be activated by synthetic agonist peptides corresponding to the tethered ligand sequence [11]. PARs are widely expressed by cells within the cardiovascular system. Both PAR-1 and PAR-2 are expressed on vascular endothelium, smooth muscle cells, and cardiomyocytes [14]. It was reported that PAR-1 but not PAR-2 is expressed by rat cardiac fibroblasts [14], [15]. However, more recent publications indicate that PAR-2 is expressed on cardiac fibroblasts of rats and mice [16], [17]. *In vitro* studies demonstrated that activation of PAR-1 or PAR-2 on rat neonatal cardiomyocytes results in a series of molecular and morphological changes that lead to hypertrophic growth of these cells [15], [18]. We have previously shown that PAR-1 contributes to cardiac remodeling after myocardial infarction by inducing eccentric hypertrophy of cardiomyocytes [19]. Recently, we demonstrated that inflammation and infarct size were reduced in PAR-2 deficient mice in an acute model of ischemia/reperfusion injury; this resulted in long-term beneficial effects reflected by a better preservation of heart function [20]. In this model, we observed reduced levels of IL-6 in the heart after injury in PAR-2 deficient mice [20]. In addition, PAR-2 stimulation leads to MCP-1 expression in endothelial and epithelial cells [21], [22].

In the current study, we investigated the mechanism by which PAR-2 contributes to hypertrophic growth of cardiomyocytes in vitro. Furthermore, we determined the effect of cardiomyocyte-specific overexpression of PAR-2 on heart remodeling and function. Finally, we used an in vivo mouse model of myocardial infarction, induced by permanent occlusion of coronary artery, to further determine the effect of PAR-2 deficiency on the long term heart remodeling.

## Materials and Methods

### Mice

PAR-2^+/−^ mice were backcrossed at least 11 generations onto a C57Bl/6J background and bred to generate PAR-2^−/−^ and PAR-2^+/+^ littermate mice [23]. Mice overexpressing PAR-2 on cardiomyocytes were generated by construction of a transgene that contained the cardiomyocyte-specific α-myosin heavy chain (αMHC) promoter and the mouse PAR-2 cDNA. Briefly, a 1.2-kbp DNA fragment containing the coding sequence of mouse PAR-2 was cloned into a vector containing the αMHC promoter (kindly provided by Dr. F. Naya [Boston University]). Next, an 8.5-kbp *Not*I fragment, containing the αMHC-promoter, the mouse PAR-2 coding sequence, and the human growth hormone polyA sequence, was purified and injected into the pronucleus of fertilized mouse embryos (C57Bl/6J genetic background) by The Scripps Transgenic Core Facility (La Jolla, CA). Transgenic mice were identified by PCR using primers specific for the human growth hormone (hGH) polyA sequence (forward-5′-AAC CAA GCT GGA GTG CAG TGG CAC-3′ and reverse-5′-AAG GAG GGT AGA TA CCT GAG ATT-3′). Terminal tissue collection was performed on mice under isoflurane anesthesia with additional cervical dislocation. The animal study was in line with the guidelines and approved by the Office of Animal Care and Use at the University of North Carolina - Chapel Hill (IACUC ID 10-069) and complied with National Institute of Health guidelines.

### Isolation and culturing of rat neonatal cardiomyocytes

Neonatal rat cardiomyocytes were isolated using a commercial isolation kit (Worthington, Lakewood, NJ) based on the method by Toraason et al. [24]. Cardiomyocytes were separated from non-myocytes by discontinuous Percoll density gradient centrifugation and cultured as described [25]. To analyze the effects of PAR-2 activation on intracellular signaling and gene expression, cells were starved for 48 hours and stimulated with PAR-2 AP (150 µM SLIGRL, Tocris Bioscience, Ellisville, MO) or control peptide (LSIGRL) for the indicated times. To induce hypertrophy, cells were stimulated for 72 hours with PAR-2 AP (150 µM SLIGRL) or control peptide (150 µM LSIGRL, Tocris Bioscience) under serum-free conditions. Cells were also pre-incubated for 30 minutes with PD98059 (10 µM) or SB203580 (10 µM) to inhibit the activation of MEK1 and p38 MAPKs, respectively [26], [27].

### Isolation and culturing of mouse embryonic cardiomyocytes

Cardiomyocytes from hearts of embryonic (E14) WT (C57Bl/6) or αMHC-PAR-2 mice were isolated as described [27], [28]. For αMHC-PAR-2 mice, each embryo was genotyped. Cardiomyocytes of the same genotype were combined and seeded in 24 well cell culture dishes [28]. An enriched cardiomyocyte population was prepared by the pre-plating method [28]. Changes in murine cardiomyocyte size were analyzed 72 hours after stimulation with 200 µM PAR-2 AP or 200 µM control peptide as well as MEK1 and p38 inhibitor as described above.

To analyze cytokine release, cells were treated with PAR-2 AP (200 µM) or control peptide (200 µM) for 24 hours and MCP-1 and IL-6 release into the supernatant were analyzed by specific Duo-Set IC Kits (R&D Systems, Minneapolis, MN) [27] and adjusted for the total cell protein concentration.

### Analysis of cardiomyocyte hypertrophic growth

To determine changes in cell surface area, rat and mouse cardiomyocytes were visualized with a Leica inverted microscope and surface area was quantified by imaging the complete boundary using digitized image analysis software (Image J, version 1.21). After stimulation for 72 hours, 5 frames per dish were captured at ×20 magnification and the cell surface of the cardiomyocytes was averaged for each frame; in total 45 to 65 cells were analyzed per treatment [15], [29]. In addition, mRNA expression of ANF and BNP was analyzed using real time PCR as described below.

### Analysis of ERK1/2 and p38 phosphorylation

Phosphorylation of ERK1/2 and p38 MAPKs was analyzed by ELISA using Duo-Set IC Kits (R&D Systems) [20]. Cells from 12 well plates were lysed in 200 µL ice cold lysis buffer containing 1 mM EDTA, 0.5% Triton X-100, 5 mM NaF, 1 M urea, 1 mM activated sodium orthovanadate, 2.5 mM sodium pyrophosphate, 10 µg/mL leupeptin, 10 µg/mL pepstatin, 100 µM PMSF, 3 µg/mL aprotinin in PBS, pH 7.2–7.4 (Sigma Aldrich). All further steps were performed according to the manufacturer's instructions. Data were normalized with the total ERK1/2 protein expression (Duo-Set IC Kit, R&D Systems).

### Real-time PCR

Total mRNA from mouse hearts was reverse transcribed into cDNA and analyzed by real-time PCR using RealMasterMix and realplex^2^ Mastercycler (Eppendorf AG, Hamburg, Germany). Primers were designed for the SYBR-green method to prevent genomic DNA amplification and have been previously published [30] (atrial natriuretic factor (ANF) 5′-CAT CAC CCT GGG CTT CTT CCT and 5′-TGG GCT CCA ATC CTG TCA ATC-3′; B-type natriuretic peptide (BNP) 5′-GCG GCA TGG ATC TCC TGA AGG-3′and 5′-CCC AGG CAG AGT CAG AAA CTG-3′; Collagen III 5′-TGG TTT CTT CTC ACC CTT CTT C-3′ and 5′-TGC ATC CCA ATT CATCTA CGT-3′; connective tissue growth factor (CTGF) 5′-GCA TCT CCA CCC GAG TTA-3′ and TTG ACA GGC TTG GCG ATT-3′; transforming growth factor (TGFβ1) 5′-GAC GTC ACT GGA GTT GTA CGG-3′ and 5′-GCT GAA TCG AAA GCC CTG T-3′; TGFβ3 5′-TTG AGC TCT TCC AGA TAC TTC G-3′ and 5′-TTC TTG CCA CCT ATG TAG CG-3′; αMHC 5′-TCA TTC CCA ACG AGC GAA A-3′ and 5′-GCC GGA AGT CCC CAT AGA GA-3′; βMHC 5′-GAT GGA CAA TCC CCT GGT CAT-3′ and 5′-CCG AAA GTC CCC ATA GAG AAT-3′) The expression of hypoxanthine-guanine phosphoribosyltransferase (HPRT 5′-GTG GTG AAA AGG ACC TCT CG-3′ and 5′-TGA AGT ACT CAT TAT AGT CAA GGG GA-3′) was used as internal control. To analyze the expression of MMP-2, MMP-3, MMP-8, MMP-9, TIMP-1, MCP-1, IL-1β and IL-6 we used predesigned probe sets (Integrated DNA Technologies, Coralville, IA). Variations in loading were adjusted using GAPDH mRNA expression.

### Histology

Fibrosis was assessed on formalin-fixed, paraffin-embedded heart sections stained with Masson's Trichrome [25].

### Echocardiography

Echocardiography on conscious mice was performed using a VisualSonics Vevo2100 system (VisualSonics, Toronto, ON) as previously described [20], [27].

### Northern blot analysis

Samples from mouse hearts were collected, frozen in liquid nitrogen and stored at −80°C. Total mRNA was isolated using Trizol reagent (Invitrogen, Carlsbad, CA) [31], [32]. Levels of PAR-2 mRNA and GAPDH mRNA were determined by Northern blotting as previously described [19].

### Myocardial infarction model - permanent ligation of the left anterior descending (LAD) coronary artery

Male mice were anesthetized with pentobarbital (45 mg/kg), intubated, and ventilated with a small rodent ventilator (Harvard Apparatus, Holliston, MA) at a rate of 110 cycles/minute with a tidal volume of 2 ml/minute and a positive end-expiratory pressure of 2 cmH_2_O. A left side thoracotomy was performed, and the pericardium was incised. Myocardial infarction was then induced through permanent ligation of the LAD coronary artery with an 8-0 silk suture proximal to its bifurcation from the main stem. The incision was subsequently closed with a 5-0 silk suture. Mice were then allowed to recover in a temperature-controlled environment. After surgery, mice were administrated with post-operative dose of buprenorphine every 12 hours for 2 days. Mice were closely monitored and all efforts were made to minimize suffering. Four weeks later heart function was analyzed using echocardiography as described above. In addition, cardiac troponin I plasma levels were analyzed from the separate sets of sham and LAD artery occluded animals 24 hours after surgery by using a highly sensitive mouse cardiac troponin I ELISA kit (Life Diagnostics, West Chester, PA) as recently described [27] to analyze initial cardiac injury.

### Statistical analysis

All statistical analyses were performed using GraphPad Prism (version 5.0; GraphPad Software Inc., La Jolla, CA). Data are represented as mean ±SEM, unless otherwise indicated in figure legends. For 2-group comparison of continuous data, 2-tailed Student's *t* test was used. For multiple-group comparison, normally distributed data were analyzed by 1- or 2-way ANOVA and were Bonferroni corrected for repeated measures over time. A *p*-value ≤0.05 was regarded as significant.

## Results

### Inhibition of ERK1/2 and p38 MAPKs reduces PAR-2 induced hypertrophic growth of rat neonatal cardiomyocytes

**Figure 1:**
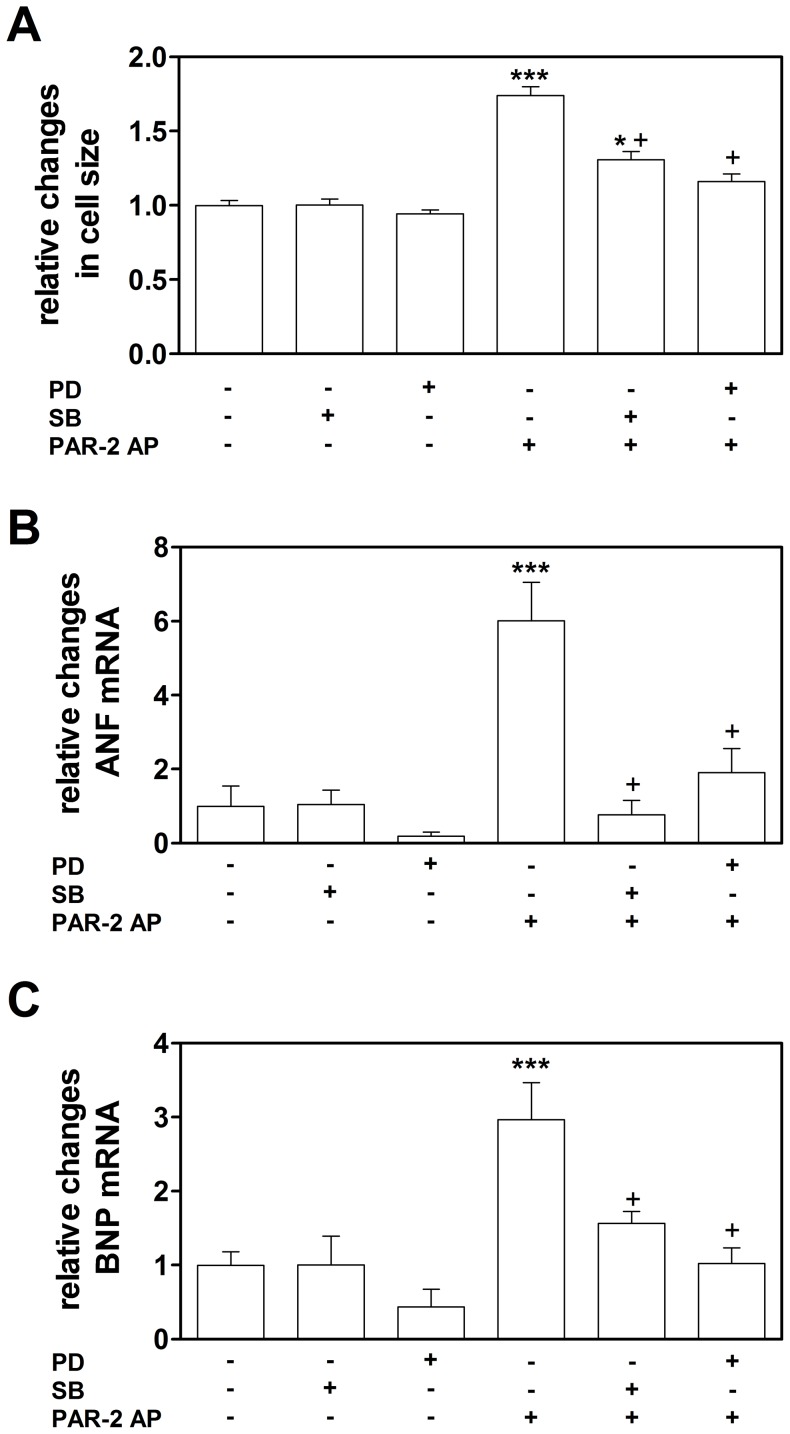
Activation of PAR-2 leads to ERK1/2 and p38-dependent cardiomyocyte hypertrophy *in vitro*. A: Changes in the total cell area of murine cardiomyocytes were analyzed after 72 hours of stimulation with PAR-2 AP in the presence or absence of MEK1 or p38 inhibitors (9–12 separate wells and 45–60 cardiomyocytes per condition, averaged from two independent cardiomyocyte isolations). **B–C**: Expression of ANF and BNP in cardiomyocytes 72 hours after PAR-2 AP stimulation in the presence or absence of MEK1 or p38 inhibitors (N = 5–8 per condition). * *p*<0.05 vs control cells; *** *p*<0.001 vs control cells; + *p*<0.05 vs PAR-2 AP treated cells without MAPK inhibitor.

Consistent with the previous study by Sabri and colleagues [15], we found that stimulation of rat neonatal cardiomyocytes with PAR-2 agonist peptide (SLIGRL, 150 µM) led to ERK1/2 and p38 MAPK phosphorylation (Figure S1A) and hypertrophic growth measured by increased ANF and BNP mRNA expression and total cell area (Figure S1B–D). Importantly, the PAR-2- mediated increase in cardiomyocyte size was attenuated by inhibition of either the ERK1/2 or the p38 MAPK pathways (Figure S1C and D). Similar to these observations, stimulation of mouse embryonic cardiomyocytes with PAR-2 agonist peptide (SLIGRL, 200 µM) also led to hypertrophic growth measured by increased ANF and BNP mRNA expression and total cell area (Figure 1A–C). Furthermore, inhibition of either ERK1/2 or the p38 pathways significantly reduced hypertrophic growth of mouse embryonic cardiomyocytes (Figure 1A–C). These data indicate that activation of these two pathways is required for PAR-2 induced hypertrophic growth of both rat and mouse cardiomyocytes.

### Generation of transgenic mice with cardiomyocyte-specific PAR-2 overexpression

**Figure 2:**
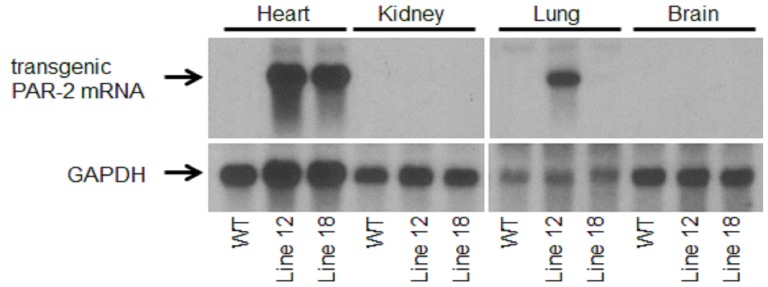
Heart specific PAR-2 overexpression. Northern blot analysis of PAR-2 mRNA expression in different organs from αMHC-PAR-2 (line 12 and 18) and littermate controls (WT) mice. Overexposed blot demonstrating expression of endogenous PAR-2 mRNA in organs is shown on Figure S2.

To directly investigate the role of PAR-2 in the heart, we generated mice overexpressing PAR-2 on cardiomyocytes using the alpha myosin heavy chain (αMHC) promoter (αMHC-PAR-2 mice). We used this promoter previously to express PAR-1 on cardiomyocytes [19]. Germline transmission of the transgene was observed in six different lines of αMHC-PAR-2 mice and four of them (lines 11, 12, 18 and 32) demonstrated a significant increase in the heart weight to body weight (HW∶BW) ratio by 2–3 months of age compared to control mice. We observed a dramatic increase in HW∶BW ratio in αMHC-PAR-2 mice line 11 (10.0±0.39 vs. 4.88±0.22; *p*<0.005; n = 3 per group) and line 32 (7.30±0.41 vs. 4.92±0.40; *p*<0.01; n = 3 per group) compared to wild type (WT) littermates, which was associated with premature death of these mice around 2–3 months of age. A moderate increase in the HW∶BW ratio was observed in line 12 at the age of 2 months (5.54±0.54, n = 7 vs. 4.85±0.20, n = 5; *p*<0.01) and line 18 at the age of 3 months (5.17±0.38 vs. 4.69±0.24; n = 6; *p*<0.05). Northern blot analysis demonstrated that the PAR-2 transgene was specifically expressed in the heart of αMHC-PAR-2 mice line 18 whereas the expression of the PAR-2 transgene in αMHC-PAR-2 mice line 12 was detected not only in the heart but also in the lung (Figure 2A). Therefore, we used mice from line 18 to study the long-term effect of cardiomyocyte-specific overexpression of PAR-2 on heart remodeling and function.

### Cardiomyocyte-specific overexpression of PAR-2 leads heart hypertrophy

**Figure 3:**
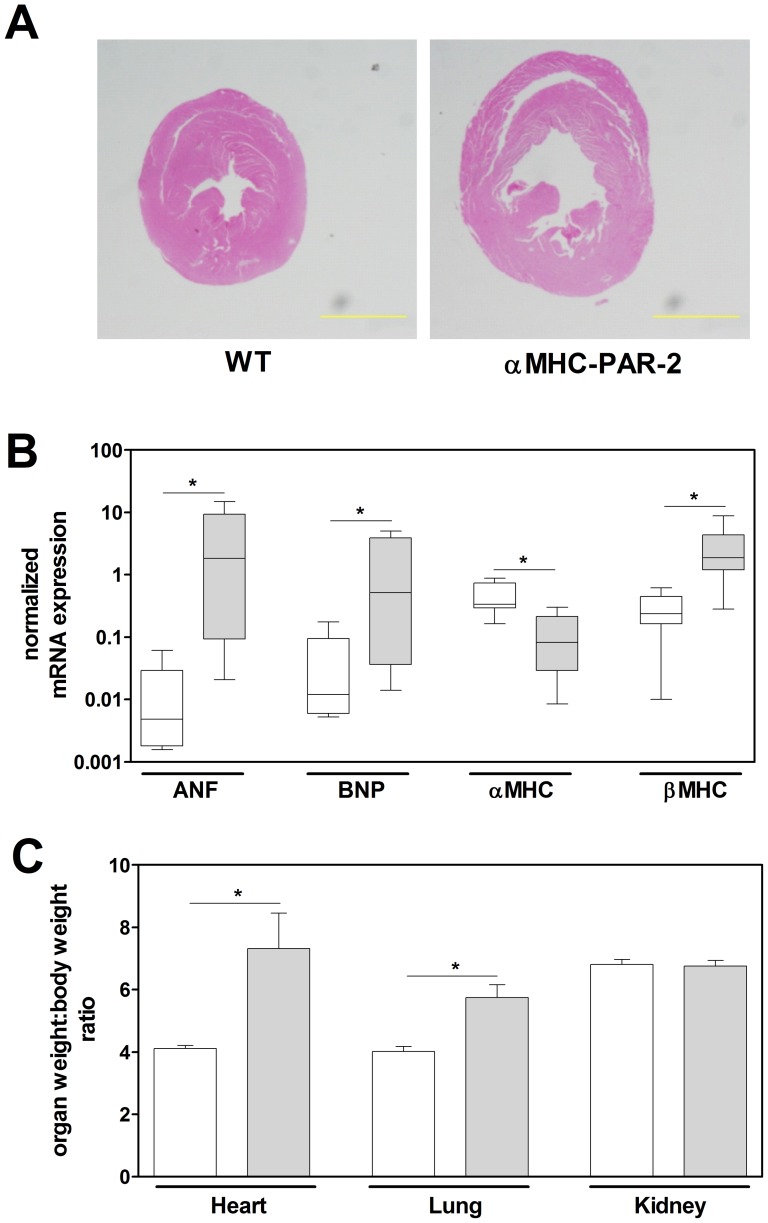
Myocardial PAR-2 overexpression leads to cardiac hypertrophy in mice. **A**: Representative cross-sections of one year old hearts from WT and αMHC-PAR-2 mice. Sections were stained with hematoxylin and eosin (Bar  = 2.0 mm). **B**: Expression of pro-hypertrophic gene quantified by real-time PCR in WT (open boxes) and αMHC-PAR-2 (grey filled boxes) at the age of one year (7–8 mice per group). **C**: The organ weight to body weight ratio was determined in one year old WT (open bars) and αMHC-PAR-2 (grey filled bars) mice (8 to 15 mice per group). * *p*<0.05.

Since we recently demonstrated that PAR-2 contributes to heart remodeling after cardiac ischemia/reperfusion injury [20] and that activation of PAR-2 leads to hypertrophic growth of cardiomyocytes *in vitro* [15], we investigated if cardiomyocyte-specific overexpression of PAR-2 induced heart hypertrophy and HF in mice. First, we analyzed the effect of the PAR-2 overexpression on 1 year old mice from line 18. Gross morphological analysis demonstrated that αMHC-PAR-2 mice had larger hearts compared to WT littermates (Figure 3A). Real-time PCR analysis showed that mRNA expression of ANF, BNP and β-myosin heavy chain (βMHC) were significantly increased, whereas mRNA expression of αMHC was significantly decreased in the hearts of αMHC-PAR-2 mice compared to the WT littermates (Figure 3B). Consistent with visibly larger hearts and altered hypertrophic gene mRNA expression, we observed an increase in HW∶BW ratio in αMHC-PAR-2 mice compared to the WT littermates (Figure 3C). In addition to heart hypertrophy, αMHC-PAR-2 mice had an increased lung weight to BW ratio (LW∶BW, Figure 3C), suggesting lung edema secondary to HF, a common co-morbidity of congestive HF. The ratio of kidney weight to BW was not changed in αMHC-PAR-2 mice (Figure 3C). The ratio of heart weight as well as lung weight to the tibia length was also significantly increased (data not shown). These data indicate that overexpression of PAR-2 in cardiomyocytes leads to heart hypertrophy in mice.

### PAR-2 dependent cardiac inflammation

**Figure 4:**
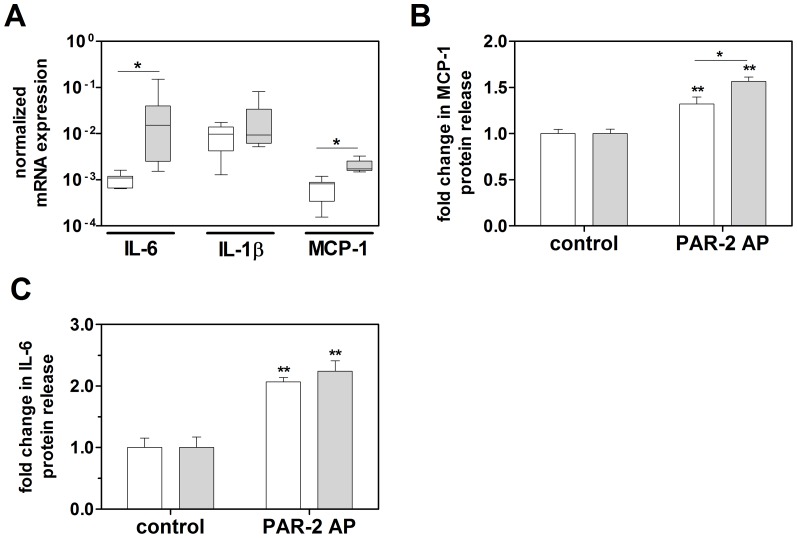
Cardiomyocyte-specific overexpression of PAR-2 results in inflammation of the heart. A: mRNA expression of inflammatory mediators in hearts of one year old WT (open boxes) and αMHC-PAR-2 (grey filled boxes) mice (7–8 mice per group). **B**: Fold changes in MCP-1 and IL-6 (**C**) protein levels in the culture supernatant from WT (open boxes) and αMHC-PAR-2 (grey filled boxes) mouse cardiomyocytes stimulated with PAR-2 AP for 24 hours. (N = 4–9 for control and N = 9–13 for PAR-2 AP). * *p*<0.05. ** *p*<0.05 vs. stimulation with scramble peptide (control).

One year old αMHC-PAR-2 mice also showed increased expression of IL-6 and MCP-1 in the heart compared to age matched littermate controls (Figure 4A). In addition, we found that PAR-2 stimulation leads to increased MCP-1 and IL-6 protein expression in embryonic murine cardiomyocytes isolated from WT mice (Figure 4B–C). Importantly, PAR-2 stimulation of cardiomyocytes isolated from αMHC-PAR-2 mice resulted in significantly higher expression of MCP-1 and also slightly higher levels of IL-6 compared to that observed in cell isolated from the hearts of WT littermate mice (Figure 4B–C). The data suggest that acute and chronic PAR-2 activation leads to cardiac inflammation.

### αMHC-PAR-2 mice develop cardiac fibrosis

**Figure 5:**
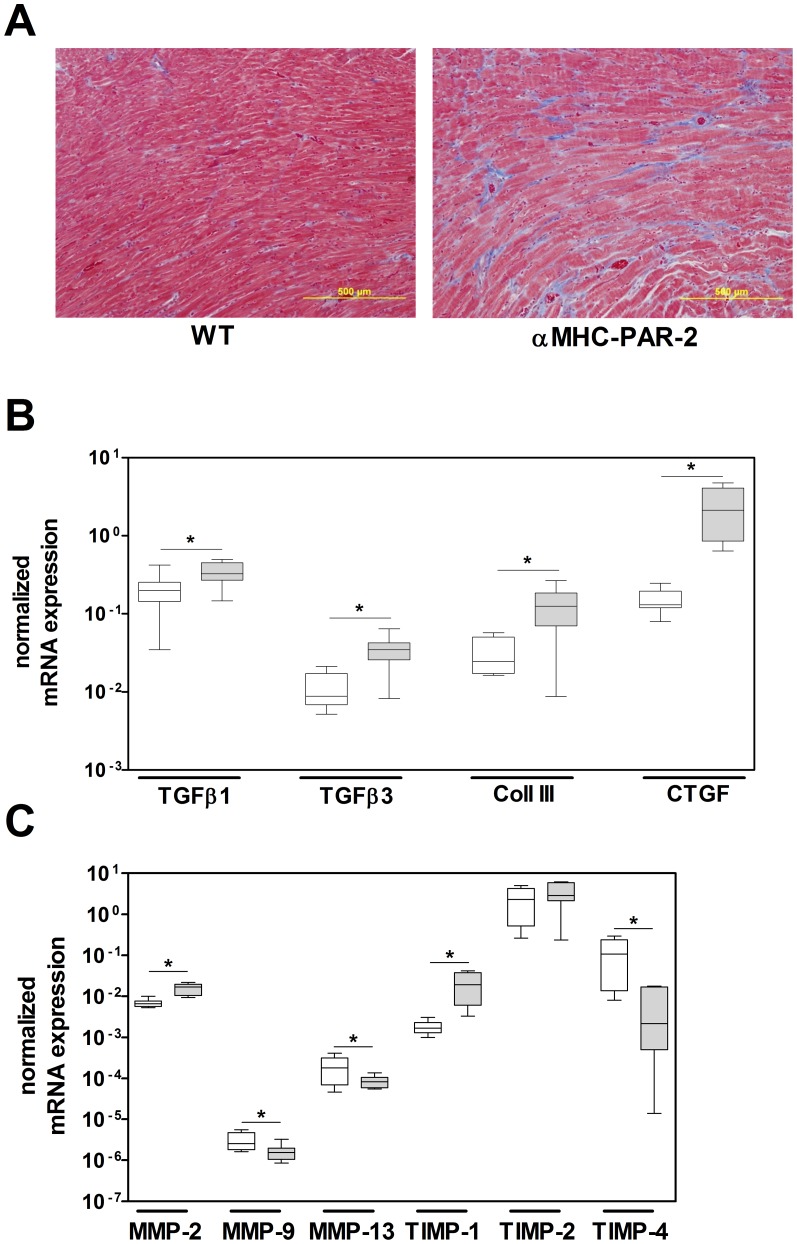
Cardiomyocyte-specific overexpression of PAR-2 results in heart fibrosis. A: Representative cross-sections of one year old WT and αMHC-PAR-2 hearts stained with Masson's Trichrome. **B–C**: mRNA expression of pro-fibrotic genes and MMPs and TIMPs in the heart of one year old WT (open boxes) and αMHC-PAR-2 (grey filled boxes) mice (7–8 mice per group). * *p*<0.05.

We showed that PAR-2 deficiency reduced cardiac fibrosis after cardiac ischemia/reperfusion injury [20]. Therefore, we investigated whether the overexpression of PAR-2 in cardiomyocytes led to cardiac fibrosis. Masson's Trichrome staining of heart sections from one year old αMHC-PAR-2 mice showed increased interstitial fibrosis (Figure 5A). Furthermore, a significant up-regulation of the mRNA expression of known pro-fibrotic genes, including TGFβ1, TGFβ3, collagen III, and CTGF, was observed in the hearts of one year old αMHC-PAR-2 hearts compared to their WT littermates (Figure 5B). Cardiac fibrosis is often associated with dysregulation of the MMP/TIMP system [3], [6]. Consistent with this notion, hearts from αMHC-PAR-2 mice exhibited increased mRNA expression of MMP-2 and TIMP-1, decreased levels of MMP-9, MMP-13 and TIMP-4 and no change in TIMP-2 compared to littermate controls (Figure 5C). These data indicate that cardiomyocyte-specific overexpression of PAR-2 resulted in pathologic heart fibrosis and remodeling due to increased matrix deposition and dysregulated MMP/TIMP system.

### Cardiac hypertrophy, inflammation and fibrosis are associated with impairment of heart function in αMHC-PAR-2 mice

Pathologic heart hypertrophy, inflammation, and fibrosis lead to heart dysfunction and HF. Therefore, transthoracic echocardiography was used to measure LV function. Consistent with the gross histological analysis, we found that the diameter and volume of the LV was increased significantly in αMHC-PAR-2 mice compared with littermate controls at 12 months of age (Table 1). Moreover, the thickness of the anterior and posterior LV wall at systole but not diastole was reduced in the αMHC-PAR-2 mice (Table 1). As expected, LV function measured by percentages of fractional shortening and ejection fraction was significantly reduced in αMHC-PAR-2 mice compared with WT littermates (Table 1).

**Table 1:** Heart function analysis by echocardiography on one year old wild-type and αMHC-PAR-2 mice.

	Wild-type	αMHC-PAR-2	*p*-value
**LVID;d** (mm)	2.90±0.35	3.44±0.45	0.01
**LVID;s** (mm)	1.80±0.33	2.62±0.55	0.001
**LVAW;d** (mm)	1.22±0.09	1.21±0.14	n.s.
**LVAW;s** (mm)	1.69±0.15	1.48±0.15	0.01
**LVPW;d** (mm)	1.23±0.16	1.16±0.13	n.s.
**LVPW;s** (mm)	1.36±0.17	1.21±0.23	0.08
**LV Vol;d** (µL)	33.07±9.71	49.99±15.30	0.01
**LV Vol;s** (µL)	10.35±4.77	26.87±13.04	0.001
**EF** (%)	69.80±6.39	48.91±11.51	0.001
**FS** (%)	38.34±5.11	24.46±6.73	0.001

LVID left ventricle internal diameter, LVAW left ventricle anterior wall, LVPW left ventricle posterior wall, Vol volume, d diastole, s systole, EF ejection fraction, FS fractional shortening.

### PAR-2 contributes to the heart remodeling after permanent occlusion of the LAD coronary artery

**Figure 6:**
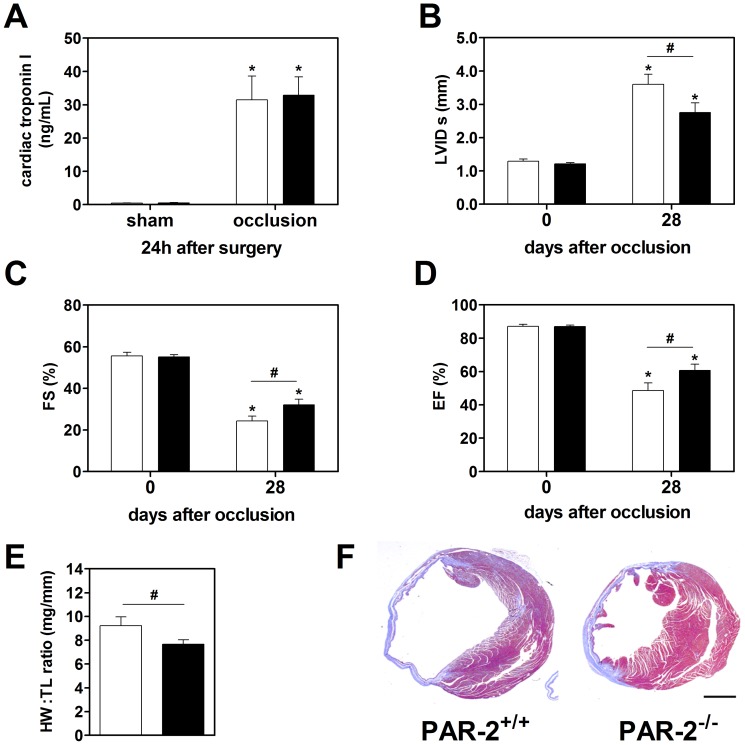
PAR-2 contributes to heart remodeling after permanent LAD occlusion. **A: **Cardiac troponin I plasma levels, as marker for cardiac injury, in sham and LAD artery occluded WT (open boxes) and PAR-2^−/−^ (black boxes) mice 24 hours after surgery (N = 4 for sham and N = 10–13 for LAD). **B**: Systolic left ventricular internal diameter (LVIDs) before and 4 weeks after occlusion of the LAD artery measured by echocardiography. Changes in heart function calculated as (**C**) fractional shortening (FS) and (**D**) ejection fraction (EF) before and after LAD artery occlusion. **E**: Cardiac hypertrophy shown as ratios of the heart weight to tibia length (HW∶TL) 28 days after permanent LAD artery occlusion. (N = 8–13 per group) **F**: Representative cross-sections of hearts from PAR-2^+/+^ and PAR-2^−/−^ mice 4 weeks after LAD occlusion stained with Masson's Trichrome. (Bar  = 1.0 mm). * *p*<0.05 vs. day 0 within the same genotype; # *p*<0.05.

To further explore the role of PAR-2 in heart remodeling independent of reperfusion injury, we used a mouse model of heart failure induced by a permanent occlusion of LAD coronary artery. First we analyzed the myocardial infarction 24 hours after permanent occlusion in PAR-2^+/+^ and PAR-2^−/−^ mice. Plasma levels of cardiac troponin I were significantly increased in both groups of mice compared to the levels observed in sham operated mice (Figure 6A). There was no difference between PAR-2^+/+^ and PAR-2^−/−^ mice indicating that PAR-2 does not contribute to the initial ischemic injury in this model. Four weeks after permanent occlusion, we used echocardiography to analyze heart remodeling and function in PAR-2^+/+^ and PAR-2^−/−^ mice. As shown in Figure 5, occlusion of the LAD resulted in significant dilation of LV and dramatic reduction of heart function. Importantly, both these parameters were significantly attenuated in PAR-2^−/−^ mice compared to PAR-2^+/+^ mice (Figure 6B–D). Heart weights of PAR-2^+/+^ mice were increased compared to PAR-2^−/−^ mice 4 weeks after LAD occlusion (208.6±16.1 mg vs. 172.4±8.2 mg, *p*<0.05). Since PAR-2^−/−^ mice body weights were slightly lower than PAR-2^+/+^ mice (27.98±0.56 g vs. 29.63±0.95 g, *p* = 0.12) whereas the tibia length were equal between the groups (22.66±0.18 mm vs. 22.55±0.10 mm, PAR-2^+/+^ vs. PAR-2^−/−^, *p* = 0.57), we used tibia length to calculate heart weight∶tibia length ratio. PAR-2 deficient mice exhibited reduced heart hypertrophy compared to PAR-2^+/+^ mice as demonstrated by lower heart weight∶tibia length ratios (Figure 6E). Representative cross-sections of the hearts from the PAR-2^+/+^ and PAR-2^−/−^ mice 4 weeks after LAD occlusion are shown in Figure 6F.

## Discussion

In this study, we demonstrated that cardiomyocyte-specific overexpression of PAR-2 led to pathologic heart hypertrophy associated with cardiac fibrosis. Pathologic remodeling of the heart in αMHC-PAR-2 mice was accompanied by increased ANF, BNP and βMHC expression and decreased αMHC expression. Importantly, BNP is a strong predictor of cardiac hypertrophy and dysfunction in both mouse models and in humans [24]. During heart hypertrophy, an initial increase in LV wall thickness is usually followed by wall thinning and dilatation of the LV chamber [5]. Echocardiography analysis revealed that the diameter and volume of the LV were significantly increased, whereas the thickness of LV walls was significantly reduced at systole but not diastole in αMHC-PAR-2 mice. Moreover, we observed a significant decrease in the heart function in αMHC-PAR-2 mice compared to littermate controls. These data indicate that cardiomyocyte-specific overexpression of PAR-2 results in pathologic heart remodeling which leads to systolic HF in mice.

Hypertrophic growth of cardiomyocytes is one of the processes that contribute to heart remodeling. Activation of PAR-2 *in vitro* leads to the hypertrophic growth of rat cardiomyocytes and increased phosphorylation of ERK1/2 and p38 [15]. However, the role of these MAPKs in PAR-2 induced cardiomyocyte hypertrophy has not been investigated. We demonstrated that inhibition of these two MAPK pathways significantly attenuated PAR-2-mediated growth of both rat neonatal and mouse embryonic cardiomyocytes *in vitro*. These data suggest that activation of PAR-2 might contribute to heart remodeling, in part, via MAPK-dependent hypertrophic growth of cardiomyocytes.

Dilated cardiomyopathy caused by pathologic hypertrophy is often associated with inflammation and fibrosis [3], [7], [8]. Cardiomyocyte-specific overexpression of PAR-2 resulted in cardiac fibrosis. The fibrotic area and levels of TGFβ, collagen III, and CTGF mRNA expression were increased in the hearts of αMHC-PAR-2 mice. In addition, mice with PAR-2 overexpression on cardiomyocytes exhibited increased cardiac inflammation seen as elevated IL-6 and MCP-1 expression. Furthermore, we demonstrated that stimulation of PAR-2 on cardiomyocytes leads to increased expression of MCP-1 *in vitro*. MCP-1 signaling leads to aberrant cardiac fibrosis and induction of HF [7], [10]. Importantly, it has been shown that inflammation influences cardiac fibrosis [7], [8]. Increased expression of MCP-1 caused changes in the balance between matrix synthesis and degeneration by interacting with the MMP/TIMP system [6], [7], [9]. End stage failing human hearts show increased expression of MMP2 and TIMP-1, and a decrease MMP-9 expression [33], [34], which we also observed in one year old αMHC-PAR-2 mice. Our data imply that chronic PAR-2 activation on cardiomyocytes induces the release of pro-fibrotic mediators, such as MCP-1, which stimulate cardiac fibroblasts leading to pathologic heart fibrosis.

Tissue factor (TF), the primary initiator of coagulation cascade is constitutively expressed by cardiomyocytes. We have previously shown that TF not only maintains heart hemostasis [35] but also contributes to myocardial infraction [36] and heart hypertrophy induced by increased PAR-1 signaling in cardiomyocytes [34]. This indicates that the TF-dependent signaling cascade plays an important role in the heart during both physiological and pathological conditions. Therefore, PAR-2 activation in the heart may occur by either the TF∶FVIIa or the TF∶FVIIa∶FXa complex [37]. Indeed, treatment of mice with active-site inhibited FVIIa attenuated inflammation and myocardial injury after ischemia/reperfusion [38]. This suggests that the TF∶FVIIa complex contributes to inflammation and cardiomyocytes injury, possibly through PAR-2 signaling. However, this does not rule out the potential effect mediated by TF∶FVIIa-dependent thrombin generation. Another potential PAR-2 activator in the heart might be mast cell tryptase [14]. Mast cells are present within the myocardium, and mast cell deficiency is associated with attenuation of cardiac remodeling and HF after injury [39], [40]. Mast cells also contribute to cardiac inflammation by expressing IL-6 [39], and MCP-1 causes mast cell degranulation. Furthermore, increased mast cell numbers, degranulation and tryptase release leads to increased MMP2 expression/activity and MCP-1 expression [21], [41], [42] providing a positive feedback loop.

We have previously demonstrated that the reduced pathologic remodeling of the heart observed in PAR-2 deficient mice after ischemia/reperfusion injury was associated with attenuation of inflammation, oxidative stress and significant reduction of myocardial infarction [20]. Importantly, the size of the initial infarct affects the extent of heart remodeling [43]. To determine if PAR-2 contributes to the heart remodeling via mechanisms other than reducing the initial infarct size, in our present study we used a mouse model of heart failure induced by a permanent occlusion of the LAD coronary artery. Since we have previously shown that PAR-2 deficiency had no effect on the topography of heart coronary vessels and the size of the area at risk in a mouse model of ischemia/reperfusion injury [20], we expect that permanent occlusion of LAD artery should result in the similar initial myocardial infarction in both PAR-2^+/+^ and PAR-2^−/−^ mice, caused by ischemia. Consistent with this assumption, plasma levels of cardiac troponin I, a well-established marker of myocardial injury, where the same in PAR-2^+/+^ and PAR-2^−/−^ mice. However, despite a similar initial myocardial infarction, PAR-2^−/−^ mice displayed reduced dilatation of LV and better preservation of heart function four weeks after permanent occlusion of the LAD coronary artery compared to PAR-2^+/+^ mice. These data indicate that PAR-2 can also directly modulate pathologic remodeling of the heart independently of its previously demonstrated role in the ischemia/reperfusion-mediated myocardial injury. Our observation is in line with the recent data showing an association of cardiac PAR-2 expression with increased cardiac inflammation and reduced heart function in patients with dilated cardiomyopathy [44].

One limitation of our study is that the phenotype of αMHC-PAR-2 mice is the result of overexpressing PAR-2. It has been previously demonstrated that overexpression may lead to the generation of non-specific effects. For example, cardiomyocyte-specific overexpression of green fluorescent protein results in HF [45]. On the other hand, cardiomyocyte-specific overexpression of various genes has significantly contributed to our understanding of heart diseases, and the results obtained from overexpression studies have been confirmed by complementary studies using knockout mice. For example, cardiomyocyte-specific overexpression of either wild type or constitutively active forms of G_αq_ induced dilated cardiomyopathy [46], [47], whereas mice with cardiomyocyte-specific deletion of G_αq_/G_α11_ are resistant to ventricular hypertrophy induced by pressure overload [48].

In contrast to our studies with PAR-2 deficient and αMHC-PAR-2 mice, it has been reported that activation of PAR-2 with a PAR-2 agonist peptide has a beneficial effect in both ex vivo and in vivo models of heart ischemia/reperfusion injury [49]–[51]. The protective mechanism involved vasodilation of coronary vessels, mediated by activation of PAR-2 on endothelial cells [51]. Similar discrepancy between treatment with PAR-2 agonist peptide and PAR-2 deficiency has been observed in the mouse model of colitis induced by intrarectal injection of trinitrobenzene sulfonic acid [52]. Interestingly, PAR-2 plays different roles in different organs subjected to ischemia/reperfusion injury. For example, PAR-2 deficiency increases the infarct volume in the brain [53], has no effect on kidney function [54] and reduces infarct size in the heart [20]. Furthermore, in a mouse model of Alzheimer disease PAR-2 signaling had opposite effects in different cell types within the brain [55]. These apparently contrasting results strongly suggest that PAR-2 mediated effects may be not only organ but even cell type-specific. Therefore, it is possible that after myocardial infarction the activation of PAR-2 on endothelial cells may be protective, whereas PAR-2 signaling on other cell types, such as cardiomyocytes or infiltrating leukocytes may be detrimental. Another possible explanation could be the fact that PAR-2 is differentially activated by tethered versus soluble ligands, such as an agonist peptide [56]. These two types of ligands differentially bind and stabilize different conformations of the receptor, leading to the activation of distinct subsets of signaling pathways [56]. A better understanding of the cell type- and ligand-specific responses of PAR-2 after myocardial infarction is needed.

In conclusion, our study suggests that PAR-2 contributes to the pathogenesis of heart hypertrophy and failure. Further studies investigating the effectiveness of specific PAR-2 inhibitors in various mouse models of heart hypertrophy and failure are warranted and will validate if PAR-2 is a good target to attenuate heart failure.

## Supporting Information

Figure S1 **Activation of PAR-2 leads to ERK1/2 and p38-dependent rat neonatal cardiomyocyte hypertrophy** ***in vitro*** **. A:** Activation of ERK1/2 and p38 signaling pathway in cardiomyocytes in response to PAR-2 agonist peptide (PAR-2 AP, 150 µM). (N = 5 each time point). **B**: Expression of ANF and BNP in cardiomyocytes after 72 h of PAR-2 AP stimulation. (N = 8–11) **C**: Changes in the area of cardiomyocytes were analyzed after 72 h of stimulation with PAR-2 AP in the presence or absence of MEK1 or p38 inhibitors. (45–65 cardiomyocytes per condition, averaged from two independent cardiomyocyte isolations). **D**: Representative pictures of cardiomyocytes 72 h after stimulation with PAR-2 AP (SLIGRL) alone or in combination with ERK1/2 (PD) or p38 (SB) inhibitors. * *p*<0.05 vs control cells; *** *p*<0.001 vs control cells; + *p*<0.05 vs PAR-2 AP treated cells without MAPK inhibitor.
(TIF)

Figure S2 **Heart specific PAR-2 overexpression.** Northern blot analysis of PAR-2 mRNA expression in different organs from αMHC-PAR-2 (line 12 and 18) and littermate controls (WT) mice. Arrow head indicates endogenous expressed PAR-2 mRNA.
(TIF)
