# Supplementary figures and images for: Protease Activated Receptor-2 Contributes to Heart Failure
Source: PLoS One. 2013 Nov 27;8(11):e81733. doi: 10.1371/journal.pone.0081733 (PMC3842269; doi:10.1371/journal.pone.0081733)

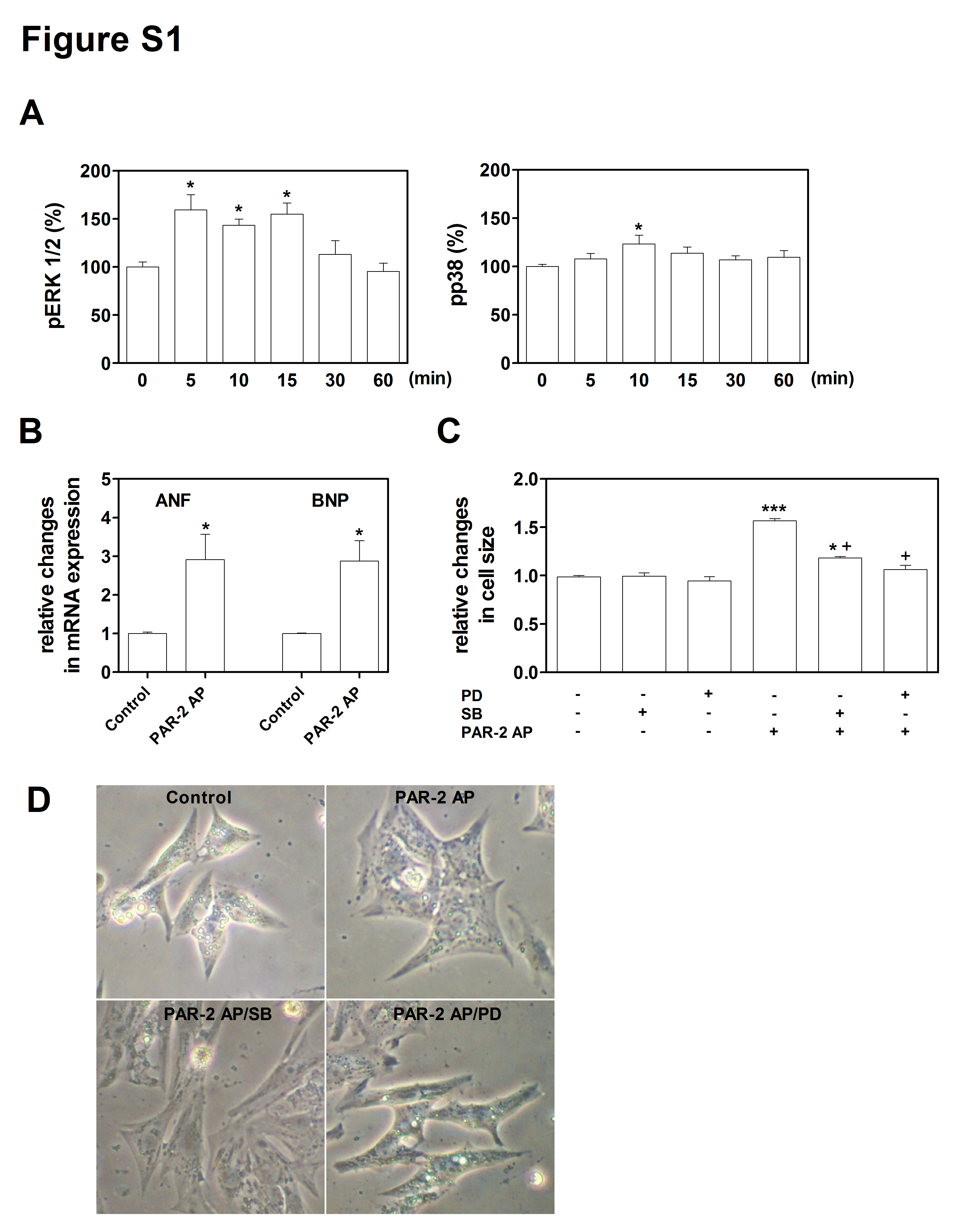

Supplement: Figure S1 — Activation of PAR-2 leads to ERK1/2 and p38-dependent rat neonatal cardiomyocyte hypertrophy in vitro . A: Activation of ERK1/2 and p38 signaling pathway in cardiomyocytes in response to PAR-2 agonist peptide (PAR-2 AP, 150 µM). (N = 5 each time point). B: Expression of ANF and BNP in cardiomyocytes after 72 h of PAR-2 AP stimulation. (N = 8–11) C: Changes in the area of cardiomyocytes were analyzed after 72 h of stimulation with PAR-2 AP in the presence or absence of MEK1 or p38 inhibitors. (45–65 cardiomyocytes per condition, averaged from two independent cardiomyocyte isolations). D: Representative pictures of cardiomyocytes 72 h after stimulation with PAR-2 AP (SLIGRL) alone or in combination with ERK1/2 (PD) or p38 (SB) inhibitors. * p<0.05 vs control cells; *** p<0.001 vs control cells; + p<0.05 vs PAR-2 AP treated cells without MAPK inhibitor. (TIF) [file pone.0081733.s001.tif]

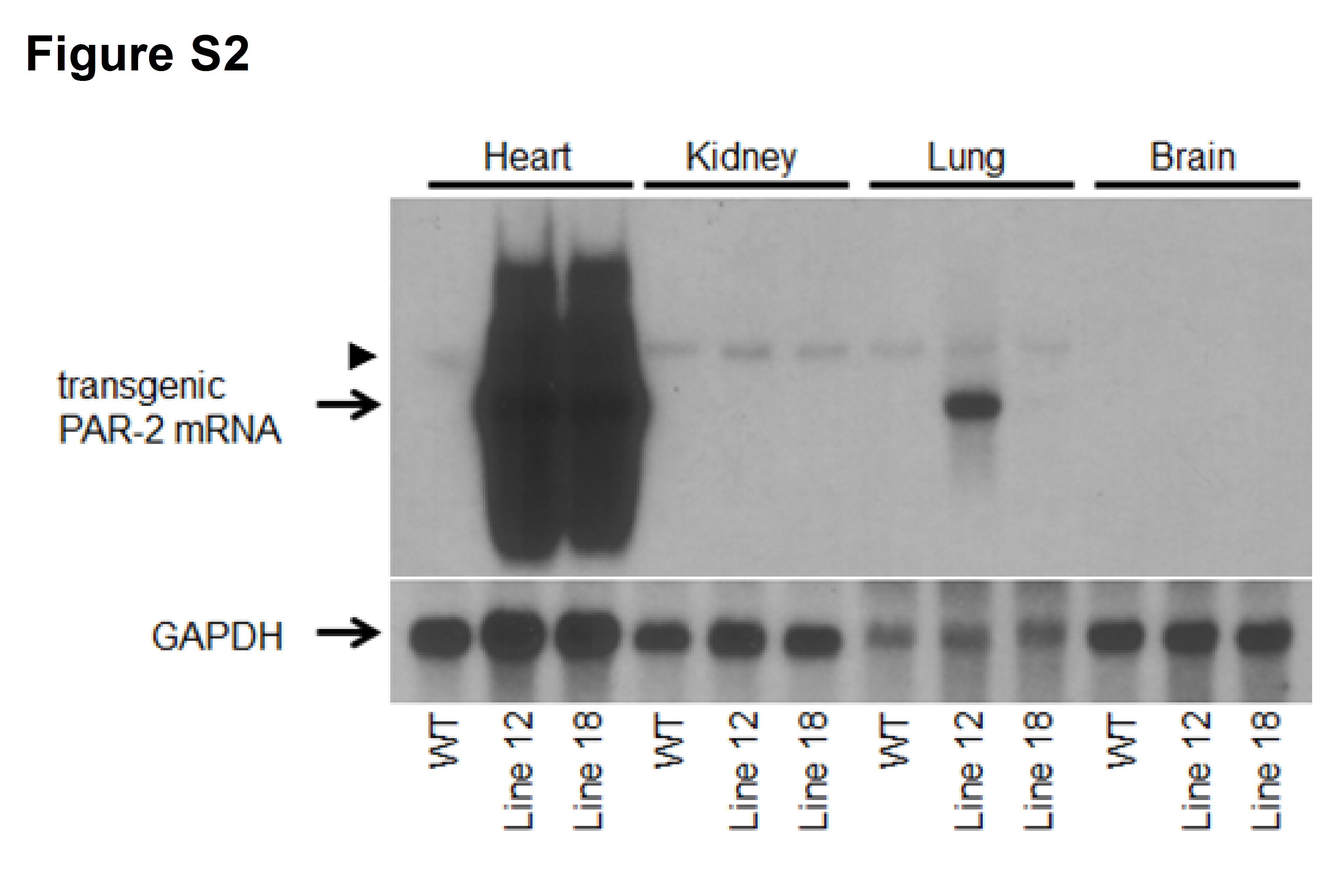

Supplement: Figure S2 — Heart specific PAR-2 overexpression. Northern blot analysis of PAR-2 mRNA expression in different organs from αMHC-PAR-2 (line 12 and 18) and littermate controls (WT) mice. Arrow head indicates endogenous expressed PAR-2 mRNA. (TIF) [file pone.0081733.s002.tif]
